# Wrist Vascular Biometric Recognition Using a Portable Contactless System

**DOI:** 10.3390/s20051469

**Published:** 2020-03-07

**Authors:** Raul Garcia-Martin, Raul Sanchez-Reillo

**Affiliations:** University Group for ID Technologies (GUTI), University Carlos III of Madrid (UC3M), Av. de la Universidad 30, 28911 Leganés, Madrid, Spain; rsreillo@ing.uc3m.es

**Keywords:** vascular biometric recognition, wrist vein recognition, contactless dataset, identification, pattern recognition, infrared camera, non-contact devices, Scale-Invariant Feature Transform (SIFT^®^), Speeded Up Robust Features (SURF^®^), Oriented FAST and Rotated BRIEF (ORB)

## Abstract

Human wrist vein biometric recognition is one of the least used vascular biometric modalities. Nevertheless, it has similar usability and is as safe as the two most common vascular variants in the commercial and research worlds: hand palm vein and finger vein modalities. Besides, the wrist vein variant, with wider veins, provides a clearer and better visualization and definition of the unique vein patterns. In this paper, a novel vein wrist non-contact system has been designed, implemented, and tested. For this purpose, a new contactless database has been collected with the software algorithm TGS-CVBR^®^. The database, called UC3M-CV1, consists of 1200 near-infrared contactless images of 100 different users, collected in two separate sessions, from the wrists of 50 subjects (25 females and 25 males). Environmental light conditions for the different subjects and sessions have been not controlled: different daytimes and different places (outdoor/indoor). The software algorithm created for the recognition task is PIS-CVBR^®^. The results obtained by combining these three elements, TGS-CVBR^®^, PIS-CVBR^®^, and UC3M-CV1 dataset, are compared using two other different wrist contact databases, PUT and UC3M (best value of Equal Error Rate (EER) = 0.08%), taken into account and measured the computing time, demonstrating the viability of obtaining a contactless real-time-processing wrist system.

## 1. Introduction

Nowadays, biometric recognition is a trendy technology that affects everyone’s safety and privacy to a greater or lesser extent. In this sense, and according to Vascular Biometric Recognition (VBR), the lack of non-contact commercial and research systems observed in the state-of-the-art has been the motivation behind this work in order to contribute to the reduced social and market integration of this technology. As is known, a contactless vascular biometric system, as facial, iris, or voice recognition systems, provides essential improvements to the user in hygiene and usability but also increases the difficulty for researchers of preprocessing, feature extraction, and feature matching in the verification/identification process. In previous research [1], a portable contactless image capture device for VBR was implemented. In the current study, this capture device is integrated (processing and storing a novel contactless algorithm and database) in order to obtain and analyze a complete contactless VBR system.

The use of the wrist area or wrist vein modality avoids the palm vein modality pattern (Fujitsu©) [2] and the finger vein modality pattern (Hitachi©) [3]. In addition, the use of this area could be considered, for future researches, in combination with other biometric research systems or techniques like Electrocardiogram (ECG) [4] or even biomedicine solutions like [5].

### 1.1. Related Work

It is important to note that, as far as it is known, there are no well-integrated and well-known commercial systems on the market based on the wrist vein modality. However, there are several studies in the research stage, as is exposed in the state-of-the-art of wrist Vascular Biometric Recognition (VBR) summarized in Table 1. It is divided into three units: dataset, capture device, and software algorithms. As it can be extracted from Table 1 and as far as it is known, there are only limited recent works, and there is only one public database for wrist vein modality: PUT [6] (50 subjects × 2 wrists × 4 samples × 3 sessions = 1200 images, 1100 genuine intraclass or mated comparisons and 108,900 impostor interclass or non-mated comparisons). This database is used in several works, e.g., [7], that also presents a complete and updated state-of-the-art of wrist VBR.

The rest of the works presented in Table 1 use two privately-distributed databases: UC3M [8] and Singapore [9]. Other less extensive works, e.g., [10], which are not presented in the table, use private databases collected with their own-designed system, as it is the case of the present study.

As far as is known, the cameras used mounted CCD sensors and LED type illumination with a wavelength of approximately 850 nm (considered the best near-infrared value for VBR).

It is essential to point out that all these databases require physical contact between the subject and the hardware part of the system, which reinforces the motivation discussed previously.

According to the recognition algorithms, all the studies follow the traditional recognition process against the trendy deep learning methods: preprocessing, feature extraction algorithm, and feature matching algorithm based on distances or machine learning techniques.

The process always begins with the preprocessing and enhancement of the near-infrared (NIR) images. The starting point is usually monochromatic images whose vein patterns are enhanced, for better definition and visualization, in the following order: contrast increase (e.g., histogram equalization), noise reduction (filters), binarization and skeletonization (e.g., Zhang and Suen [11]). Then, the task could continue (only in [9] in Table 1), with the extraction of the Region of Interest (ROI). For feature extraction, several techniques are applied: minutiae extraction, as the own algorithm discussed in [8]; feature extraction base on Local Binary Pattern (Dense Local Binary Pattern), [12]; Hessian matrix, [13]; and convolution approach, [14]. The matching algorithms are based on distance (Hausdorff distance [9,14] and own minutiae algorithm [8]) and cross-correlation comparison [13].

Traditional Machine Learning methods for matching are only employed in [12] (Support Vector Machines).

Computing time for the entire software algorithm is given in the latest works, [12,13], revealing the evolution of biometric systems nowadays. As the results of the proposed system indicate (Section 3, Section 3.2.2), computing time is a critical variable in the integration of real-time biometric systems.

The performance for all works indicated in Table 1, based on the Equal Error Rate (EER), varies between 0.14% and 2.27%. These values should be considered reduced enough, but it is important to remark, as it has been mentioned, all the devices required physical contact with the users, fixing the wrist position and easing the recognition task. The images obtained are extremely invariant in scale and orientation, which translate into really high biometric performance, as it is demonstrated in the current work, with entirely similar features extracted. In this sense, a new scale-orientation-invariant algorithm is presented in the current study.

Another important factor noticed in the capture devices, due to the contact feature, is the immunity to the environmental or external light. This light does not reach the capture device due to the closed space between the camera and the wrist. Again, as a result, the similarity between the images is improved and, of course, the recognition performance. A non-contact system, as it is demonstrated in this paper, is affected by the external light conditions despite the extra capture light illumination. These two factors are the goals to overcome in order to improve wrist VBR recognition and obtain contactless devices.

### 1.2. Contributions

The main goal of the work presented in this paper is to obtain and test a complete, low-cost, real-time, contactless vascular biometric system based on wrist vein recognition. For this purpose, the capture algorithm, TGS-CVBR^®^, and the capture device exposed in [1] are integrated and used in the current study to collect a contactless database (UC3M-CV1). Then, a new scale-orientation-invariant software algorithm, based on Scale-Invariant Feature Transform (SIFT^®^), Speeded Up Robust Features (SURF^®^), and Oriented FAST and Rotated BRIEF (ORB), is proposed and tested on the database: Preprocessing and Identification Software for Contactless Vascular Biometric Recognition (PIS-CVBR^®^). The present work is summarized in the supplementary video material.

## 2. Materials and Methods

**Figure 1:**
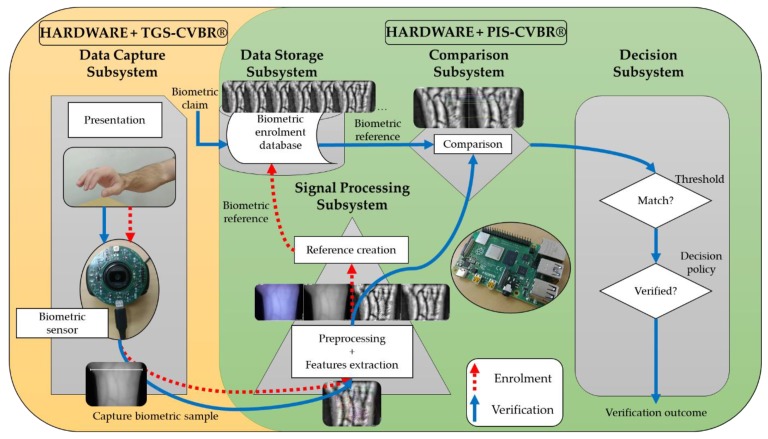
Components of the experimental wrist VBR system.

The experimental procedure, material, and methods are summarized in Figure 1 (expired on ISO/IEC 19795-1:2019 [29]). For the research and implementation of a complete wrist VBR contactless system, the following elements have been defined:

(1) Hardware: present in all the subsystems of Figure 1 (capture, storage, signal processing, comparison, and decision).
(2) Software algorithms: divide into two software algorithms TGS-CVBR^®^ and PIS-CVBR^®^. The first one is in charge of the data capture (yellow and left side of Figure 1) and the second one takes care of the and storage, preprocessing, comparison and decision tasks (green and right side of Figure 1). Combining these two algorithms a final system is obtained.

The following units of this section detail the procedure for obtaining these two elements: hardware (capture, processing, and storage devices) and software algorithms (TGS-CVBR^®^: capture algorithm and PIS-CVBR^®^: storage, signal processing, comparison, and decision).

### 2.1. Hardware: Capture, Storage and Processing Devices

The hardware only implemented as a capture device in [1] was integrated and used in the current study as a capture, processing, and storage system. It consists of three parts: near-infrared camera (sensor for the capture) near-infrared Printed Circuit Board (PCB, LED illumination) and small computer (processor and storage). The camera selected and modified was the commercial USB webcam Logitech^®^ HD Webcam C525 [30]. For the infrared lighting, a PCB with eight infrared LEDs (OSRAM© SFH 4715 A [31], 850 nm) was designed and manufactured. In the current work, the small computer Raspberry^®^ Pi 4 Model B [32] was used, instead of the Raspberry^®^ Pi 3 Model B [33] of [1], for VBR processing and database storage.

### 2.2. Software Algorithms

As was mentioned, the software algorithm is divided into two fragments. The first one, Three-Guideline Software for Contactless Vascular Biometric Recognition (TGS-CVBR^®^, presented in [1] only as a capture algorithm), is used to guide users on how to position the wrist in the database collection (image capture and visualization). The second one, Preprocessing and Identification Software for Contactless Vascular Biometric Recognition (PIS-CVBR^®^), is the recognition algorithm.

#### 2.2.1. TGS-CVBR^®^

**Figure 2:**
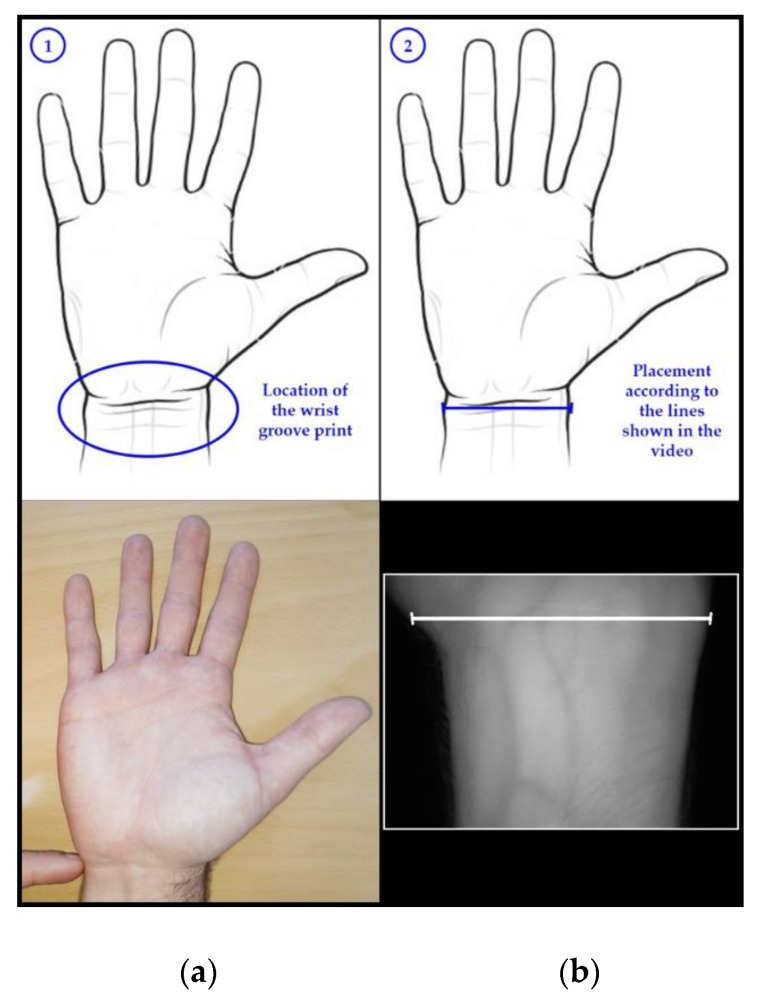
Three-Guideline Software for Contactless Vascular Biometric Recognition (TGS-CVBR^®^), wrist positioning steps (based on [1]). (**a**) Step 1: the location of the wrist groove line. (**b**) Step 2: match of the wrist groove print and the guideline.

The real-time video of the camera capture (640 × 480 resolution) was displayed on a monitor together with the three fixed guidelines, as is shown in Figure 2 (step 2, right side). This algorithm provided feedback to the user on how he/she was positioning the wrist and was used for database collection (UC3M-CV1, in this case) and user recognition (combined with PIS-CVBR^®^). The guidelines were useful because they fixed the user’s wrist, obtaining scale-orientation-invariant images in order to improve the recognition algorithm task: the largest horizontal guideline sets the wrist orientation, and the two smaller guidelines establish the distance between the wrist and the camera.

This software was developed using Python™ 3.4.2 due to the quick and easy way to access to the USB camera and the well-integration of the language with deep learning libraries, in order to be used in future works.

The user should follow the steps shown in Figure 2:

1. Locate the wrist groove print or mark.
2. Align/match it with the guide trace displayed.

#### 2.2.2. PIS-CVBR^®^

After the database collection (experimental process explained in the later section), the next step was to recognize the user: authentication/verification (1:1 user comparison) or identification (1:N user comparison). For this purpose, Preprocessing and Identification Software for Contactless Vascular Biometric Recognition (PIS-CVBR^®^) is proposed in this paper. It was divided into three parts or steps: preprocessing, feature extraction, and feature matching.

This software has been also developed using Python™ 3.4.2.

##### Preprocessing

**Figure 3:**
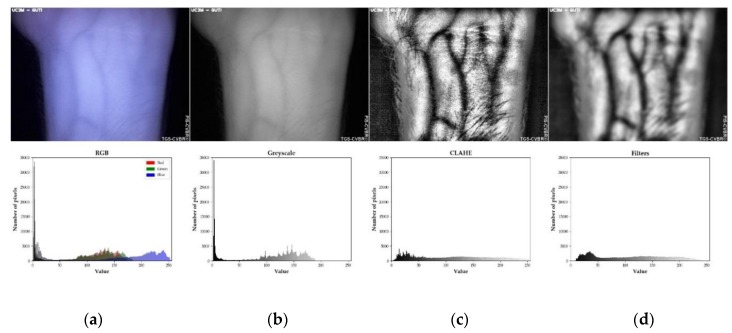
Preprocessing and Identification Software for Contactless Vascular Biometric Recognition (PIS-CVBR^®^): Preprocessing steps for User 0. Example images (above) and their histograms (below): (**a**) RGB image. (**b**) Image after greyscale conversion. (**c**) Image after greyscale conversion and Contrast Limited Adaptive Histogram Equalization (CLAHE) algorithm. (**d**) Image after greyscale conversion, CLAHE algorithm, and filtered by Gaussian filter, Median filter, and Averaging (11 × 11 kernel).

The main goal of preprocessing was to enhance, normalize, and define the vein patterns in other to extract the features later on. This process is summarized in Figure 3. The infrared RGB images were captured in 640 × 480 resolution and “.jpg” compressed format (Figure 3a) with TGS-CVBR^®^ and the modified camera (RGB camera). The first step, RGB to greyscale (monochromatic image with values from 0, black, to 255, white) transformation, is shown in Figure 3b.

In order to obtain a higher contrast between veins and the rest living tissue, the adaptive histogram equalization technique Contrast Limited Adaptive Histogram Equalization (CLAHE) [20] was used (Figure 3c). To reduce the high-frequency noise (salt-and-pepper and Gaussian noise in this case) generated by this algorithm and the camera sensor, several low-pass software filters were employed (Figure 3d) in the following order: Gaussian filter, Median filter, and Averaging filter. The kernel size of all of them was 11 × 11. This was the last step of the preprocessing task.

Finally, it is important to remark that in this paper, and at this moment, the ROI extraction was not considered required for this software. However, it would probably improve system performance and is a step to contemplate in the future.

##### Feature Extraction

For the extraction of unique features from the wrist vein patterns, three scale-orientation-invariant algorithms for homography have been used and tested: Scale-Invariant Feature Transform (SIFT^®^) [34], Speeded Up Robust Features (SURF^®^) [35] and Oriented FAST and Rotated BRIEF (ORB) [36]. They have been selected and used, along with the TGS-CVBR^®^ algorithm, in order to avoid the variability of the size and orientation of the wrist area, caused by the non-contact feature.

The first algorithm, SIFT^®^, patented in 2004 [34], was based on the Harris Corner Detector, whose variant-scale features were the motivation for improvement. SIFT^®^ is a well-known algorithm due to its excellent performance but also to its high computing time. In order to reduce this time, SURF^®^ was patent in 2006 [35]. Finally, the ORB algorithm, a fusion of the modified FAST and BRIEF algorithms, was published in 2011 as an open-use and a faster alternative.

**Figure 4:**
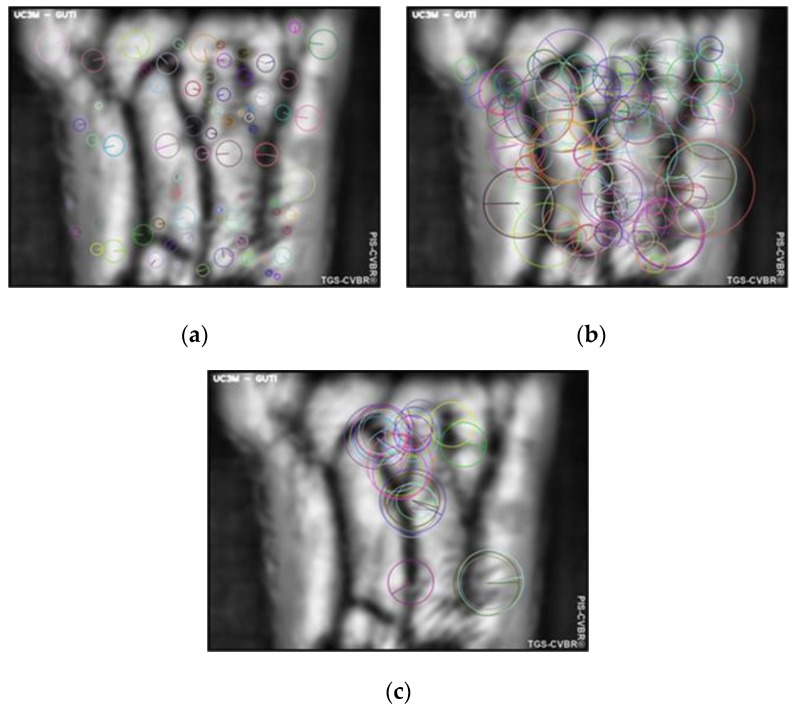
PIS-CVBR®: Feature extraction for User 0. Scale and orientation of the 100 key points extracted with the three algorithms used: (**a**) Scale-Invariant Feature Transform (SIFT®). (**b**) Speeded Up Robust Features (SURF®). (**c**) Oriented FAST and Rotated BRIEF (ORB).

In VBR, only SIFT^®^ has been used in the wrist variant, in [37]. However, in the current study, these three algorithms have been compared, and also, with a contactless dataset. After the preprocessing, the performance of the feature extraction (100 key points) for each algorithm, with scale and orientation, is shown in Figure 4a–c, respectively.

##### Feature Matching

For the feature or key points matching, two algorithms were used:

1. Brute Force Matcher (BFM): for the descriptors of the features extracted with ORB.
2. Fast Library for Approximate Nearest Neighbors (FLANN) [38]: for the descriptors of the features extracted with SURF^®^ and SIFT^®^.

**Figure 5:**
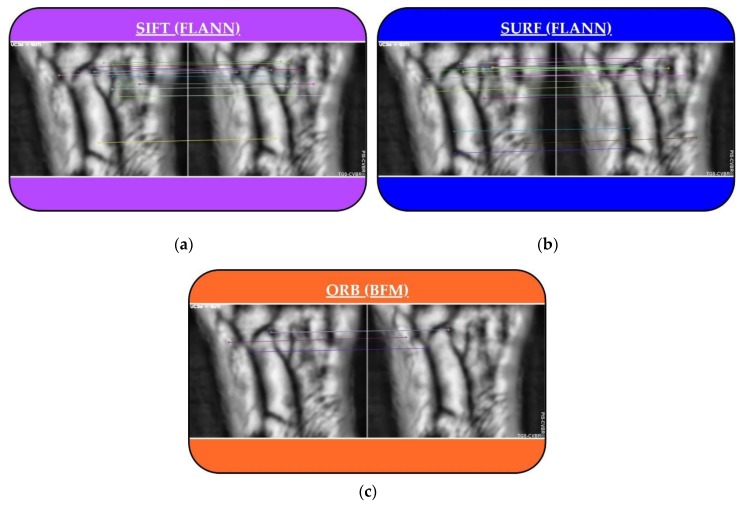
PIS-CVBR^®^: feature matching for User 0. Correct matching points for two samples of User 0 with the three feature extraction algorithms: (**a**) SIFT^®^ (with Fast Library for Approximate Nearest Neighbors (FLANN)). (**b**) SURF^®^ (with FLANN). (**c**) ORB (with Brute Force Matcher (BFM)).

The matching between the wrist pattern image of one user (User 0) and real-time video capture was taken and shown, for the two matching algorithms, in Figure 5.

The BFM and the FLANN algorithms provided distances between the features matched. These distances are similarity values between matched features or key points. For the BFM, the Hamming distance was selected. A higher value of distance means that the points were more separated, i.e., they were less similar. To decide if these matched points are suitable, the Lowe’s ratio test [34] was used for FLANN (SIFT^®^ and SURF^®^), and a simple distance score value was set for BFM (ORB). The result of performance per each algorithm is discussed in Section 3.2.2.

**Figure 6:**
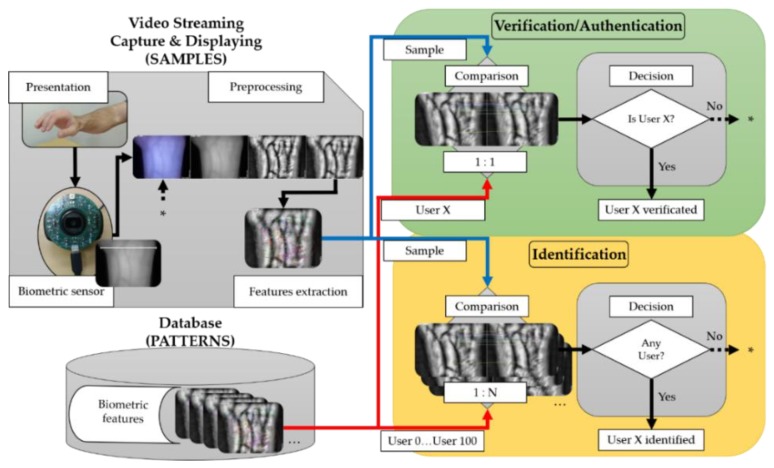
TGS-CVBR^®^ and PIS-CVBR^®^ union: Real-time authentication and identification process.

So as to obtain a real-time authentication and identification system, analyzing the computational performance of the proposal software algorithms, TGS-CVBR^®^ and PIS-CVBR^®^ are combined. Figure 6 shows and summarized the authentication and verification process made in this work.

**Figure 7:**
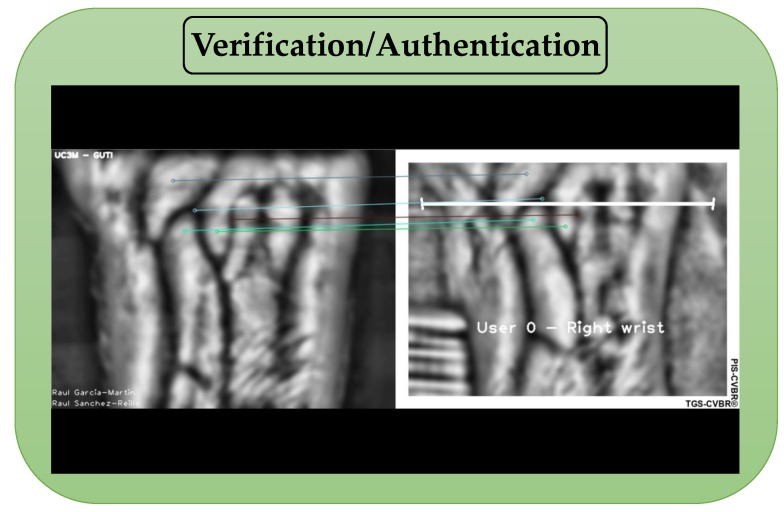
TGS-CVBR^®^ and PIS-CVBR^®^ union: User 0 real-time authentication (screenshot). Unique user image pattern (**left** side) comparison with video (**right** side) using (SIFT algorithm, 7–8 FPS). In the video, the word “User” refers to the subject, and the wrist is predefined (User 0 = Subject 0 and Right wrist, User 1 = Subject 0 and Left wrist).

For the authentication or verification task (green block in Figure 6, 1:1 user comparison), the unique user image pattern (User X extracted from the database) was compared with the real-time video capture (samples), i.e., the features extracted from the image (Figure 7, left) were matched with the features extracted from the streaming video (Figure 7, right). Please, check the supplementary video material for better comprehension.

**Figure 8:**
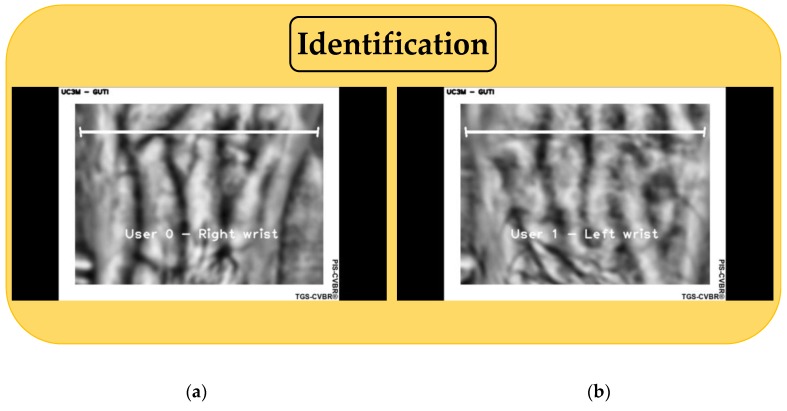
Final system software: User 0 and User 1 real-time identification using TGS-CVBR^®^ and PIS-CVBR^®^ (SIFT algorithm). (**a**) User 0 capture. (**b**) User 1 capture.

For the identification task (yellow block in Figure 6, 1:N user comparison), once the unique features had been extracted from each user (User 0 to User 100) at the initialization of the program, they were compared with real-time video capture. Figure 8 shows two identification examples of two users. It is important to notice that according to the normative ISO/IEC 19795-1:2019 [29], this software does not identify because does not provide a rank index, R, of the number of users considered as potential candidates selected with a threshold T.

The computing performance for these two tasks is detailed in the results section, Section 3.

### 2.3. Dataset Collection: Experimental and Evaluation Procedure

The database acquired in this work was named UC3M-Contactless Version 1 (UC3M-CV1) database. It was collected with the proposed TGS-CVBR^®^, and the hardware described previously. The two other databases detailed in Table 1 and acquired with physical contact, UC3M [8] and PUT [6], were employed in this study in order to compare, with contact and non-contact dataset, the results obtained with the software algorithms proposed: TGS-CVBR^®^ and PIS-CVBR^®^.

#### 2.3.1. Parameters

##### Subject Conditions

The UC3M-CV1 database was made of 1200 infrared greyscale 640 × 480 images captured from 100 users: both wrist of 50 subjects (25 females and 25 males) from Europe (43), America (4), Africa (1) and Asia (2) aged between 21 and 75 years (39.92 years on average, 17.74 standard deviation).

**Figure 9:**
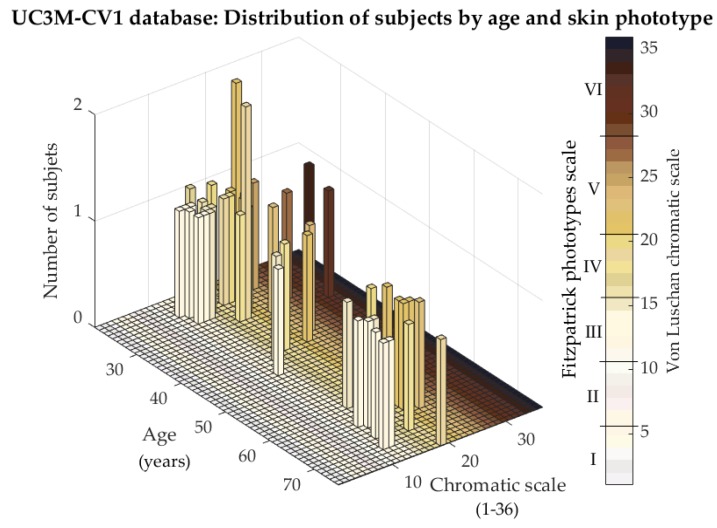
UC3M-CV1: Distribution of subject by age and skin phototype, according to the Fitzpatrick phototypes scale and the von Luschan chromatic scale.

The age and the skin color distribution, according to the Fitzpatrick phototypes scale [39] and the von Luschan chromatic scale, is shown in Figure 9. As is pretended in recent works as [40], studying different environmental (light, temperature, and humidity) and subject conditions, the idea was to introduce new concepts that may affect the vein visualization. In this case, one subject condition is reflected: skin color. It is claimed that skin damage [41] and skin pigmentation [42] do not affect the visualization of the veins in the palm and the finger vein modalities. In these areas, the melanin concentration is lower due to the thickness of the skin. However, the wrist region had slightly higher levels of melanin. These levels increased in dark-skinned subjects, and, as detected in this work, without conclusive results, they could affect the process of vein visualization. For this reason, in Figure 9, the subject phototype distribution is reflected. The chromatic scale distribution was clearly displaced to values under 21 (phototypes I to IV), and the age was mainly distributed between 20–30 and 60–70 years.

The influence of the continent-region was also another issue that is not addressed in this work but is also a factor to take into account in future researches. The origin distribution should be higher.

Six samples per session were captured for each subject wrist: 50 subjects × 2 wrists × 6 samples × 2 sessions = 1200 images. These monochromatic images have been stored in “.jpg” compressed format. More than two weeks and less than four weeks was the distance between sessions. 

The size of this dataset could be increased in future works, but it is essential to remark that it is larger in the number of sessions and samples than the UC3M dataset but smaller in the number of sessions than the PUT dataset.

##### Environmental Conditions 

The samples have been taken under uncontrol environmental conditions:

1. Temperature: Approximately 20–23 °C.
2. Humidity: Dry ambient.
3. External light: Different daytimes, places (outdoor/indoor), and external artificial lights (usually without direct sunlight).

#### 2.3.2. Collection Method

For the generation of the database, the next steps have been followed:

- The volunteers were informed of the experiment they will be part of and their rights according to the last General Data Protection Regulation (GDPR, applied since May 25th, 2018) [43]. Then, they signed the explicit consent.
- Registration of the personal data of the subject.
- Brief demonstration for the subject, following and showing it in Figure 2, on how to position the wrist correctly according to TGS-CVBR^®^.
- One operator took one capture when the user’s wrist was placed correctly. The operator helped the user (voice indications) if it detected that the subject was placing the wrist in an extremely wrong way: too far/near from the camera (not following the two small guidelines) or with an incorrect orientation (not placing the wrist grove print aligned with the largest guideline).
- The capture process was repeated, obtaining 12 samples per each subject (six samples per wrist): one session per subject. The external light conditions between the different subjects were not the same: different days at a different time in different places (outdoor/indoor).
- Two weeks after the first session, steps 4 and 5 were repeated in the second session obtaining 24 samples per each subject (12 samples per wrist) in total.

## 3. Results

In order to evaluate the different parts exposed in this paper, this section follows the structure of the previous one. The experimental evaluation procedures have been detailed.

### 3.1. Hardware: Capture, Storage and Processing Devices

**Figure 10:**
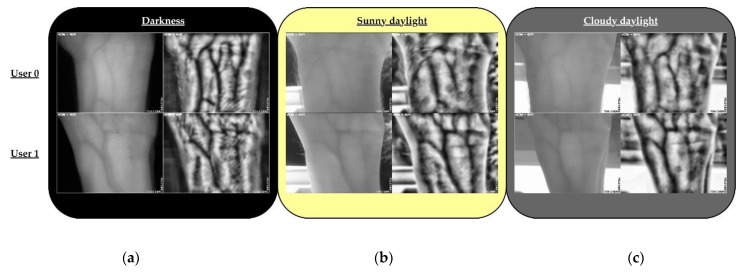
Results: outdoor external light conditions for User 0 (top row) and User 82 (bottom row). (**a**) Darkness. (**b**) Sunny daylight. (**c**) Cloudy daylight.

As was presented in the previous work [1], the NIR camera and the NIR PCB illumination provided homogeneous light distribution and good quality images, avoiding excessively bright or dark areas. In this paper, in order to evaluate the response of the capture device in different ambient light conditions, several images were taken. The main goal of this evaluation was to introduce the environmental light influence concept as a critical issue to use this type of system outdoors. Figure 10 shows the comparison of one image of the right wrist of two subjects, User 0 and User 82, in three different outdoor ambient light conditions: darkness, sunny daylight, and cloudy daylight, respectively Figure 10a–c. As a first approach, these conditions were heuristics because the luminous intensity had been not measured.

As can be seen, veins patterns were also visible and recognizable in sunny and cloudy day scenarios, considered as unfavorable light ambient conditions. However, the influence of the conditions of the scenarios is also remarkable.

In the darkness, the resulting images are quite similar, with homogenous light diffusion, to the ones obtained with the contact device used at [8]. The quality of the images was slightly lower in this work, but it is worth taking into account the reduction in size and cost of the camera and illumination. Otherwise, as has been mentioned, it is important to point out that most of the images collected for the UC3M-CV1 were taken indoor with artificial light conditions but without direct sunlight.

The processing time performance was a hardware-software relation requirement that is analyzed and discussed in the next section (Section 3.2.2, Processing-time performance).

### 3.2. Software Algorithms

#### 3.2.1. TGS-CVBR^®^

This software component is also evaluated in [1], demonstrating a reduced variation in size and orientation (illumination in consequence) of the wrists. The recognition process was improved, although the algorithms selected and used in this paper (PIS-CVBR^®^), SIFT^®^, SURF^®^, and ORB were already scale-orientation-invariant algorithms.

**Figure 11:**
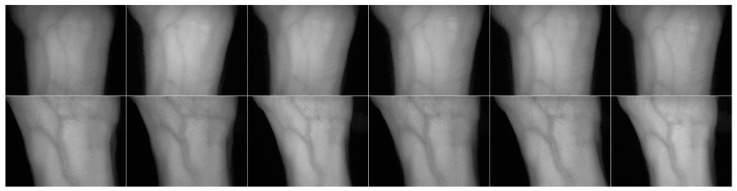
Three-Guideline Software for Contactless Vascular Biometric Recognition (TGS-CVBR^®^) images comparison for six samples of User 0 (**top row**) and User 82 (bottom row) [1].

Figure 11 shows the results of the use of TGS-CVBR^®^. The repetitiveness in the samples is evident.

In addition, although a usability test for the subjects was not realized for the collection of the UC3M-CV1 dataset, they have indicated that they felt comfortable as the sessions and sample capture have been going on. In the future, a usability test should be passed for a complete evaluation.

#### 3.2.2. PIS-CVBR^®^

The PIS-CVBR^®^, software algorithm, was analyzed, according to the normative ISO/IEC 19795-1:2019 [29], in two different ways for the three algorithms used: biometric system performance and processing-time performance. In this way, the three algorithms selected have been tested: SIFT^®^, SURF^®^, and ORB.

##### Biometric System Performance

For this purpose, the database generated, UC3M-CV1, was used. As is mandatory by the normative [29], the False Match Rate (FMR) and False Non-Match Rate (FNMR) were provided in a Detection Error Trade-Off (DET) plot (recommended). Failure-To-Enrol Rate (FTER) and Failure-To-Acquire Rate (FTAR) were unknown.

**Figure 12:**
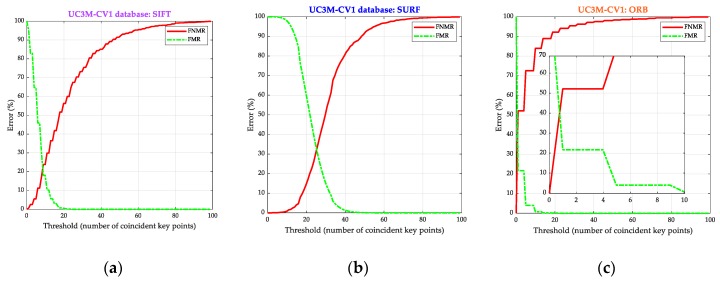
Results: biometric system performance. It is represented the percentage of error, False Match Rate (FMR) (line-dot green curve), and False Non-Match Rate (FNMR) (continuous red curve) versus the threshold of the number of coincident key points. (**a**) SIFT^®^. (**b**) SURF^®^. (**c**) ORB.

The first approach of the biometric performance obtained from the UC3M-CV1 database, Figure 12a–c shows, respectively, for each algorithm the error rate in % versus the threshold (number of coincident key points that determine the acceptance or rejection of the user). These graphics discussed the FMR and the FNMR according to the threshold for the 1100 intraclass or mated comparisons (50 subjects × 2 wrist patterns × 11 samples), and the 108,900 interclass or non-mated comparisons (100 wrist patterns × 99 wrist patterns × 11 samples) made.

The threshold, shown in the decision subsystem in Figure 1, was the number of coincident points to choose and accept a user. The green curve represents as a percentage the False Match Rate (FMR) or value of samples compared that should be rejected but are accepted by the algorithm. The red curve represents as a percentage the False Non-Match Rate (FNMR) or value of samples compared that should be accepted but are rejected by the algorithm.

These graphics anticipate the best biometric performance for SIFT^®^ and the worst for ORB, just analyzing the high value for the crossing point, EER: 21.76%, 32.29%, and 39.94% for respectively, SIFT^®^, SURF^®^, and ORB, and the integer thresholds of 9, 25, and 1.

**Figure 13:**
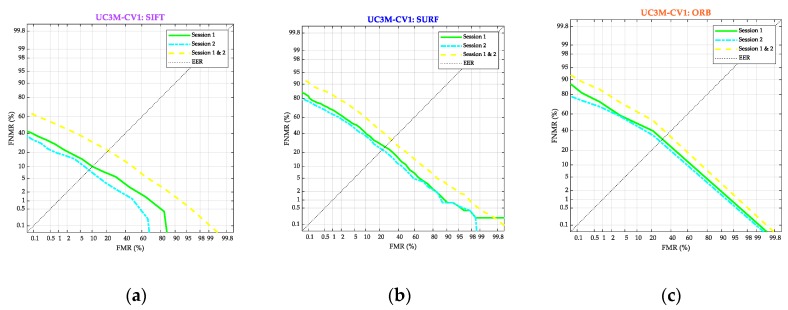
Results: Biometric system performance. Detection Error Trade-Off curve. The False Non-Match Rate is represented versus the False Match Rate. The green (continuous), cyan (line-dot), and yellow (line-line) curves are respectively for Session 1, Session 2, and the full database. (**a**) SIFT^®^. (**b**) SURF^®^. (**c**) ORB.

In order to verify this prediction, the Detection Error Trade-Off (DET) curves were obtained (Figure 13) for the collected UC3M-CV1 database and according to the three algorithms (SIFT^®^, SURF^®^, and ORB, respectively in Figure 13a–c) and the two capture sessions (Session 1 with green curve, Session 2 with cyan line-dot curve and the entire dataset with yellow gap-line curve).

The results for SIFT^®^ were confirmed as significantly better for all the sessions. The curves were clearly closer to small values for the SIFT^®^ case. It is interesting to point out that, for the three algorithms, the biometric performance improved in the second session, comparing it with the first, showing a more homogeneous placement of the wrist by the subjects in the capture. Otherwise, the curve performance for the entire UC3M-CV1 dataset gets worse. These results have been clearly treated in the discussion section.

In order to compare them with the results obtained with a physical contact database, the UC3M [8] and PUT [6] dataset have been processed with the same software algorithms. The compared results are shown below.

**Figure 14:**
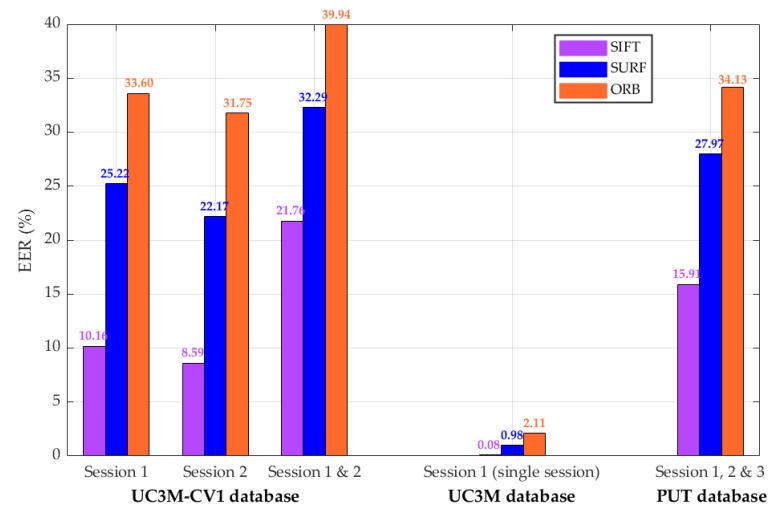
Results: Biometric system performance. Equal Error Rate (EER) obtained for each database using TGS-CVBR^®^ and PIS-CVBR^®^ with SIFT^®^, SURF^®^, and ORB algorithms.

Although the EER value is deprecated according to the normative [29], Figure 14 shows this value obtained for SIFT^®^, SURF^®^, and ORB.

The better performance for this working point for the UC3M-CV1 and the two other databases was obtained using, respectively, SIFT^®^, SURF^®^, and ORB algorithms.

For the UC3M-CV1 database and the three algorithms, the EER value is reduced in the second session (from 10.16 in Session 1 to 8.59 in Session 2, in SIFT^®^ case), most probably, as it was mentioned, due to the practice of the subject using the system. However, obtaining the results for both sessions, the full database, the EER reached inadmissible values: 21.76%, 32.29%, and 39.94%. These EERs should not be compared with the results obtained and presented in the current state-of-the-art with physical contact devices. 

For the UC3M database, the results were much better, probably due to three factors:

1. Single session: the UC3M database was collected in one session. This fact avoids subject usability variability.
2. Contact system: The system used in [8] fixed extremely the position (scale and orientation) of each user with the contact capture device (non-portable big size system). In addition, the device isolated the wrist from the external illumination conditions.
3. Images quality: For the generation of this database, probably, the quality of the images obtained was better with the usage of a dedicated industrial camera [8] (larger size and higher price).

For the PUT database, the EER values were higher than in the UC3M database but lower than in the UC3M-CV1. The capture contact system [6] obtained worse quality images but homogeneous in illumination without external light influence.

**Figure 15:**
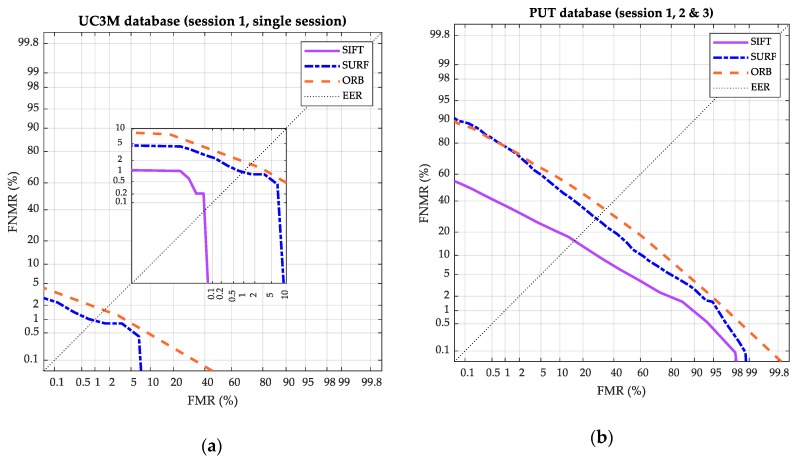
Results: Biometric system performance. DET curve. The FNMR percentage is represented versus the FMR percentage. The purple (continuous), blue (line-dot), and orange (line-line) curves are respectively for the SIFT^®^, SURF^®^, and ORB. (**a**) UC3M. (**b**) PUT.

In order to ratify these results, the DET graphics for the two other databases used are shown in Figure 15a (UC3M) and Figure 15b (PUT).

The EER obtained for the UC3M dataset with SIFT^®^, shown in the extended purple DET curve of Figure 15a, reached 0.08%. This value was significantly lower than the obtained in all the studies of Table 1 (state-of-the-art summary). In the case of the PUT dataset, the results are clearly improvable, comparing the current values with ones of the state-of-the-art. This comparison denotes the high correlation that exists between the design of the software algorithms and the datasets in which they are tested, in agreement with the way the datasets have been collected (mainly the capture device).

Finally, it is essential to remark that in all studied cases, SIFT^®^ and SURF^®^ algorithms obtained better results, but also, the computational cost was significantly higher for the key points extraction and matching, as it is analyzed in processing-time performance unit.

The results were obtained using Python™ 3.4.2. and Matlab^®^ R2019b.

##### Processing-Time Performance

**Figure 16:**
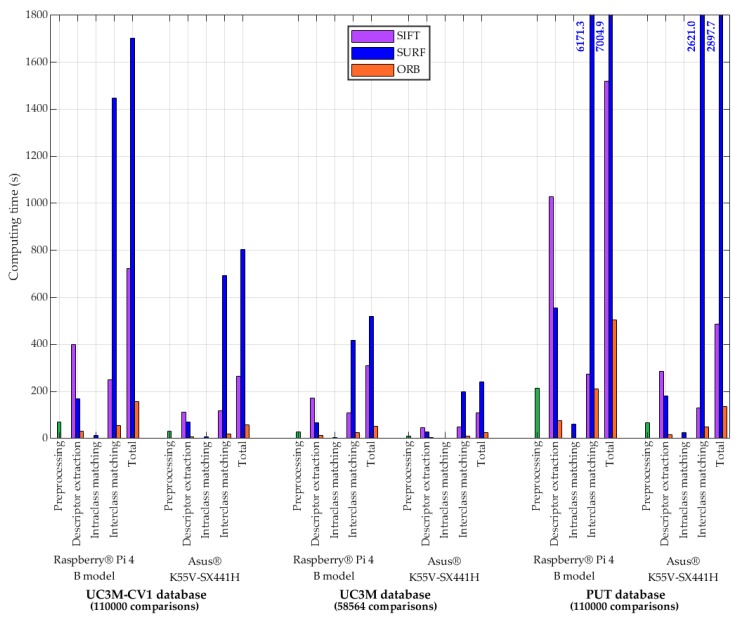
Results: Processing-time performance in seconds for each database and its computing hardware using TGS-CVBR^®^ and PIS-CVBR^®^ with SIFT^®^, SURF^®^, and ORB algorithms. Preprocessing time is in green color due to its independence with feature extraction algorithms.

Figure 16, for the three algorithms, shows the computing time spent in the completed UC3M-CV1, UC3M [8] and PUT [6] databases for the preprocessing, the feature extraction (generation of key points and its descriptors), the intraclass (mated) and interclass (non-mated) comparison (feature matching) and the total time. The hardware used for processing, Raspberry^®^ Pi 4 Model B [32], provided with 64-bit ARM-Cortex A72 (1.5 GHz, quad-core), 4 GB RAM and 128 GB external flash memory, is compared with the Asus^®^ K55V-SX441H [44] 64-bit laptop provided with Intel^®^ Core™ i7 3630QM (2.4 GHz, quad-core), 8 GB RAM and 1 TB of SSD memory. As can be seen, the processing time was considerably reduced in the laptop.

The ORB algorithm was faster in all aspects. SURF^®^ was always slower matching features than SIFT^®^, that is slower extracting descriptors. The processing time of the PUT database was higher than in the UC3M-CV1 database (same number of images, subjects, and comparisons) due to the “.bmp” image non-compressed format. It has been verified that there was no loss of information with the “.jpg” format for the algorithms used. The results were obtained using Python™ 3.4.2. and Matlab^®^ R2019b.

The processing time performance of the real-time authentication and identification system is summarized in Table 2 for the three algorithms used and the UC3M-CV1 database.

As has been indicated previously, in the processing time performance of PIS-CVBR^®^, SIFT^®^ and SURF^®^ were slow algorithms with a high computational cost. This was reflected in obtaining reduced values of Frames Per Second. As the footer of Table 2 indicates, values below 2–3 FPS were considered too low rates to obtain a real-time processing system, producing an unacceptable lag effect.

The results have been obtained using Python™ 3.4.2.

## 4. Discussion

In this work, a wrist vein non-contact capture system (hardware and software) for Vascular Biometric Recognition (VBR) has been designed, implemented, and tested. For this purpose, 1200 near-infrared images have been taken and analyzed with a novel contactless capture algorithm. According to the current state-of-the-art, this system tries to contribute to the VBR research world obtaining a system with the following remarkable main features and goals:

1. Contactless.
2. Real-time processing.
3. Portable: small size (85.60 mm × 56.5 mm × 17 mm) and weight (0.2 kg).
4. Reduced price (less than 200 $).
5. Invariant to environmental light conditions.

All these aspects have been demonstrated and fulfilled, except for the invariance to external light conditions. In order to obtain them, in the hardware part, a homogeneous NIR PCB illumination has been integrated. Two new software algorithms have been registered: TGS-CVBR^®^ and PIS-CVBR^®^. The first one fixes, in a contactless way, the orientation and the scale of the wrist, in order to avoid differences in the illumination and to ease the feature extraction process. The second one, PIS-CVBR^®^, is in charge of preprocessing (enhancing and increasing vein patterns visualization despite suboptimal environmental light conditions) and of the identification process. For the identification process, the texture based-on homography algorithms, SIFT^®^ [34], SURF^®^ [35], ORB [36] are used. These well-known algorithms are invariant to scale and orientation, a property a priori advantageous for the purpose.

In order to test the biometric and the processing time performance, a new contactless database has been generated, UC3M-CV1, with 100 users (both wrists of 50 subjects) in two sessions.

Finally, the results reflect the following conclusions:

1. The portable and cheap hardware allows obtaining homogenous illumination, avoiding dark and bright areas, although it is not completely immune to the environmental (sunlight an artificial light) conditions (Figure 10). As a required improvement, precise control of the sensor’s near-infrared wavelength sensitivity and the pass-band near-infrared filter would be essential, but probably not definitive, for the achievement of this goal. In addition, the quality of the sensor could be improved. In this sense, the results obtained with PIS-CVBR^®^ reflect that the biometric performance for the two sessions is clearly better in a separate way than for the full UC3M-CV1 database.
  According to the processing or computing time, it is thought that the small computer used, Raspberry^®^ Pi 4 Model B [32], mounts an enough powerful computing hardware for real-time processing these types of recognition software algorithms, despite the issues evinced by the slowest, SIFT^®^.
2. The TGS-CVBR^®^ fulfills its goal providing scale-orientation-invariant images, i.e., wrists with the same orientation or positioning and the same size for each user. Nevertheless, user interaction with this type of guiding-feedback algorithms, related to the biometric performance, presents an unexplored field that should be researched in the future.
3. The PIS-CVBR^®^ evinces, as it was stated in point 1, that the biometric performance is completely linked to the environmental light conditions. As far as it is known, this issue had not been addressed in other works that usually employ devices statically in a laboratory and with the sensor isolated from the external light influence. It is thought that the preprocessing step is correct due to the high and clear visualization of the vein patterns. However, the recognition results are entirely not acceptable. In the future, in order to obtain a contactless real-time-processing VBR system, all efforts will be focused on the improvement of the algorithm (biometric performance) and its execution speed (processing time performance), according to the hardware selected.

## 5. Conclusions

In this paper, a novel vein wrist non-contact VBR system has been designed, implemented, and tested. For this purpose, a contactless device has been integrated with a guiding algorithm, TGS-CVBR^®^. A novel preprocessing registered method for pattern vein definition has been created. A new non-contact database with 100 different wrists and 1200 infrared images, UC3M-CV1, has been collected. Three scale-orientation-invariant algorithms, SIFT^®^ [34], SURF^®^ [35], and ORB [36], have been tested on it and two other databases (physical contact datasets). Selecting the SIFT^®^ algorithm as the one with the best biometric performance (but worst processing time performance), the results denote the need to continue researching on wrist VBR contactless algorithms, although the improvement against the state-of-the-art results (EER = 0.08% for the UC3M database).

In the future, the lines of research will continue, firstly, with the enhancement of the system invariance against the environmental light and the integration of these devices, introduced in this work. Secondly, the biometric performance will be improved taking into account the scale and orientation of the wrist in the image strongly related to the external light influence. For this purpose, new embedded devices and, against the traditional recognition process, deep learning algorithms are being researched.

## 6. Patents

From the work reported in this paper, no patents have resulted nevertheless, two software algorithms have been registered: TGS-CVBR^®^ and PIS-CVBR^®^.

## Figures and Tables

**Table 1:** Summary of the state-of-the-art for wrist Vascular Biometric Recognition (VBR).

Title		Infrared Imaging of Hand Vein Patterns for Biometric Purposes [9] (2007)	Vascular Biometrics Based on a Minutiae Extraction Approach [8] (2011)	Spectral Minutiae for Vein Pattern Recognition [14] (2011)	A New Wrist Vein Biometric System [12] (2014)	Fast Cross-Correlation Based Wrist Vein Recognition Algorithm with Rotation and Translation Compensation [13] (2018)
**Dataset**	**Name**	Singapore (NIR, Own) [9]	UC3M (Own) [8] and Singapore [5]	UC3M [8] and Singapore [9]	PUT (Public) [6]	PUT [6]
	**Subject**	150	121	Same as UC3M and Singapore.	50	Same as PUT.
	**Wrists**	2	1 (right)	Same as UC3M and Singapore.	2	Same as PUT.
	**Samples**	3	5	Same as UC3M and Singapore.	12 (4 per session)	Same as PUT.
	**Sessions**	N/A	1	Same as UC3M and Singapore.	3	Same as PUT.
	**Total Images**	900	605	Same as UC3M and Singapore.	1200	Same as PUT.
**Capture device**	**Images Acquisition**	CCD KP-F2A Hitachi Denshi NIR camera, Hoya RM80 optical NIR 800 nm high pass filter	CCD Imaging Source DM 21BU054 camera, EYSEO TV8570 1/3” objective and B + W 52 092 and B + W 52 093 optical NIR high pass filters	Same as UC3M ^b^ and Singapore ^a^.	USB camera	Same as PUT.
	**IR Light**	LEDs (850 nm)	LEDs (880 nm): DOM 1410 (DCM system)	Same as UC3M and Singapore.	LEDs (850 nm)	Same as PUT.
	**Type**	Reflection	Reflection	Same as UC3M and Singapore.	Reflection	Same as PUT.
	**Contactless**	No	No	Same as UC3M and Singapore.	No	Same as PUT.
**Software algorithms**	**Preprocessing**	Monochromatic images, Noise reduction (Median filter + 2D Gaussian low pass filter), Normalization ([15]), Binarization (own thresholding) and Skeletonization (Zhang and Suen [15]).	Monochromatic images, Contrast increase (own histogram equalization), Noise reduction (Median filter + 2D Gaussian low pass filter), Normalization ([15]), Binarization (own thresholding) and Skeletonization (Zhang and Suen [15]).	Monochromatic images, Enhanced (Adaptive non-local means [16]), Noise reduction and edge enhancing [17]), Inversion, Binarization ([18]) and Skeletonization (fast marching algorithm [19]).	Monochromatic images, Adaptive histogram equalization [20] and Discrete Meyer Wavelet [21]	Monochromatic images, Gaussian filter and k-means++ algorithm [22]
	**ROI**	Sobel filter	No	No	No	No
	**Feature Extraction**	No	Minutiae extraction (own algorithm)	Convolution approach ([23]) and Location-Based Spectral Minutiae Representation (SML, [24]).	Dense Local Binary Pattern (D-LBP, own algorithm)	Hessian matrix
	**Feature Matching**	Hausdorff distance	Vector minutiae comparison (own algorithm)	Hausdorff distance, Modified Hausdorff (MHD) [25,26], Similarity-based Mix-matching (SMM) [27], SML correlation (SMLC, own) and SML fast rotate (SMLFR, own).	Support Vector Machines (SVMs) [28]	Cross-correlation based comparison
	**Computing time (s)**	N/A	N/A	N/A	0.771 (Windows, Matlab, i5 CPU)	0.92 (Linux, Python, i7-5930K CPU)
	**Performance**	No	EER_UC3M_ = 2.27% EER_SINGA._ = 1.63%	EER_UC3M (SMM)_ = 1.18% EER_SINGA. (SMM)_ = 0.14 % (SMM)	EER_PUT_ = 0.79%	FNMR _PUT_ = 3.75% for FMR _PUT_ ≈ 0.1%

**Table 2:** Results: final system software. Processing-time performance in Frames Per Second (FPS) for the UC3M-CV1 database and its computing hardware using TGS-CVBR^®^ and PIS-CVBR^®^ with SIFT^®^, SURF^®^, and ORB algorithms.

Hardware	Authentication (FPS, 1 User)			Identification (FPS, 100 Users)		
	SIFT^®^	SURF^®^	ORB	SIFT^®^	SURF^®^	ORB
Raspberry^®^ Pi 4 Model B	2 (*)	4–5	8	2 (*)	2–3 (*)	4
Asus^®^ K55V-SX441H	7–8	9–10	15	6	9	15

* Framerate too low to obtain a real-time processing system.

## Supplementary Materials

The following are available online at https://zenodo.org/record/3696767#.Xl-o6aj0na8, Video S1: UC3M-Wrist_Vascular_Biometric_Recognition_Using_a_Portable_Contactless_System.mp4.
